# Supplementary material for: Recent Methods for Modifying Mechanical Properties of Tissue-Engineered Scaffolds for Clinical Applications
Source: Biomimetics (Basel). 2023 May 16;8(2):205. doi: 10.3390/biomimetics8020205 (PMC10204517; doi:10.3390/biomimetics8020205)
Supplement: Supplementary file 1 [file biomimetics-08-00205-s001.zip › biomimetics-2320212-supplementary.pdf]

Supplementary Table S1 – A synopsis of each study described within this article, including the authors and publication year, material and tissue type, testing and mechanical property information, as well as a brief summary, where appropriate, of each research paper reported this far.

PCL: Polycaprolactone, HA: Hydroxyapatite, hPE: Human placental extracts, PGA: Poly-glutamic acid, Col: Collagen, HA-NFs: Hydroxyapatite nanofibres: GelMA: Methacrylated gelatin, GO: Graphene oxide, PHEMA: poly(2-hydroxyethyl methacrylate), TMP-BG: Thixotropic magnesium phosphate-based gel, SF: Silk fibroin, HyAMA: Methacrylated hyaluronic acid, PEC: polyelectrolyte complexation, SA: Sodium alginate, PrP: Platelet-rich plasma; rSF: Regenerated silk fibroin, SESM: Soluble eggshell membrane, MC: Methylcellulose, ZnO: Zinc oxide, VEGF: Vascular endothelial growth factor, dECM: Decellurised extra-cellular matrix, Ti: Titanium, PLGA: Poly-lactic-co-glycolic acid: PLA: Polylactic acid, PCS: Polymerized chondroitin sulphate, PEO: Polyethylene oxide, PES: Polyethersulfone, HA-Sr: Strontium doped hydroxyapatite, BH: Benzyl hyaluronan, BMP-2: Bone morphological protein-2, TGF-β3: Transforming growth factor-β3, SilMA: Methacrylated silk, BG: Bioglass, HyA: Hyaluronic acid, RHC: Recombinant human collagen, GAGF: Glycosaminoglycan foam, PLLA: Poly(L-lactic acid), PDA: Polydopamine, OcPh: Octacalcium phosphate, P3HB: Poly-3-hydroxybutyrate, AC-dECM: Articular cartilage decellurised extra-cellular matrix, ePTFE: Expanded Polytetrafluoroethylene, RGD: arginine-glycine-aspartate, PGS: Poly(glycerol sebacate), DTβ4: Dimeric thymosin β4, PEG: Poly(ethylene glycol), PCEC: PCL-b-PEG-b-PCL.

**Author, year, reference**	**Material Type**	**Fabrication method**	**Tissue type**	**Testing type, strain rate/force, and sample size (mm)**	**Young's Modulus in Tension (T), and/or Compression (C) (MPa)**	**Storage and Loss Modulus (kPa)**	**Failure Strain (%/%)**	**Yield stress and ultimate tensile strength (MPa)**	**Degradation: structure remaining**	**Porosity (%) and pore size (μm^2)**	**Swelling ratio**	**Applied to animal research?**	***In vivo* summary**	**Cell behaviour (*in vitro/in vivo*)**	**Mechanical performance summary**
Rezania, et al. (2022) [29]	PCL/ HA	3D printing	Bone	Compression and tension 1 mm/min Ø 80 L 1.6	237.27-340.72 (T) and 31.522–62.67 (C)	-	10-20	6.80 - 9.13	-	37 and 400	N/A	No		No significant change.	Addition of HA to PCL increased mechanical strength of scaffold, but also decreased elasticity.
Lee, et al. (2022) [117]	Gelatin /HA/ hPE	3D printing	Bone	Cyclic compression 30 mm/min H 30 W 30 D 3	0.11-0.12 (C)	-	-	-	8.83% to 9.39% after 14 days	82.3 and 402.7	N/A	Yes	The loading of hPE into the Gelatin/HA scaffold induced a superior osteogenic response compared to that of the unmodified scaffold.	The addition of hPE significantly increased cell proliferation in comparison to gelatin alone.	While hPE increased modulus slightly, the strength of the scaffold is largely insufficient to act as a substitute for bone.
Nguyen, et al. (2022) [115]	PGA/HA/ Col	3D printing	Bone	Dynamic 0.2 N/min Ø 8 L 2.5	0.062-0.272 (C)	~1.5-18.4 and ~0.187-5	40-55	0.022 – 0.111 -	76.6% to 81.9% after 63 days	~15 – 85 and 266	N/A	Yes	The Col + PGA/HA + PGA scaffold indicated the highest cell proliferation and osteogenesis. The next highest cell viability was found in the Col + PGA scaffold. The membrane-based scaffold, the cell core appeared on the surface of the membrane, with the ECM inside it. The Col scaffold showed the lowest cell viability.	Col/PGA/HA/PGA scaffold offered greatest cell proliferation, followed by the membrane combination. This suggests that HA was a key factor in cell viability.	Col/PGA/HA/PGA scaffold boasted increased mechanical strength compared to other combinations. Insufficient for bone, however.
Wang, et al. (2022) [118]	GelMA/HA-NFs	Hydrogel solution	Bone	Compression 1 mm/min Ø 8.5 L 3.5	~0.020 - 0.027 (C)	-	~65 – 78	- ~0.080 – 0.130	~20% to 42% after 56 days	- 100	~450%-550% after 35 hours	Yes	Increasing quantities of HA-NFs in the GelMA promoted a stronger osteogenic response, with 15 and 25 wt/wt% HA-NFs showing new bone deposition and blood tissue formation, compared to 0 and 5 wt/wt% which showed little new tissue formation.	Slight decrease in cell performance compared to control scaffold (GelMA)	Introduction of m-HANF to the GelMA hydrogel proportionally increased mechanical strength with increasing quantities, and marginally increased strain performance.

Tabatabee e, et al. [119] (2022)	Gelatin/ GO/ PHEMA	Freeze-drying	Bone	Compression<br>1 mm/min<br>-	9.03-42.82 (C)	-		-<br>-	~20% to complete degradation after 8 weeks	-<br>50-300	254 to 522% after 48 hours	No		Increasing GO content slightly decreased cell proliferation in comparison to control scaffold (TCP)	Minor increase in GO content (0.25 > 0.75%) significantly increased strength of PHEMA/Gel scaffold.
Ramasamy, et al. (2022) [128].	PLLA/EBNs/MWCNTs	Electrospinning	Bone	Tensile<br>1.5 mm/s<br>-	~10-40 (T)	-	60-92	~0.1-2 and 0.47-3.6	94.3-91.5% after 4 weeks	-<br>-	-	No		After 21 days of culture, it was observed that, in comparison to the plain PLLA scaffold, cell viability was increased on those scaffolds containing EBNs, further for the scaffold containings MWCNTs, and again for the hybrid of these materials.	In terms of mechanical strength, yield, and UTS performance, strength was improved in comparison to the pure PLLA scaffold by the inclusion of MWCNTs, improved further by inclusion of EBNs, and even further with a combination of these materials. Strain performance was somewhat reduced in the latter two scaffolds.
Jiawei, et al. (2022) [129]	Sugarcane/HA	Delignification and mixing	Bone	Compression<br>1 mm/min<br>10 x 10 x10	0.75-1.11 (curved surface area (CSA)) (C)<br>4.82-14.6 (longitudinal) (C)	-	6.5-10 (CSA)	~0.05-0.15 and ~0.3-0.9	-	68-76.1 and 10-350	N/A	No		No significant differences were observed in cell proliferation rate between scaffold types, however cells seemed to proliferate well across all scaffolds considered.	For both CSA and longitudinal directions, native sugarcane stem offered higher moduli and UTS values than any of the modified stems. However, strain performance in the longitudinal direction was marginally improved in the modified stems.
Tevlek, et al. (2021) [136]	PLLA/Cell sheet	Electrospinning	Bone	Tensile<br>-<br>Ø 30<br>L 1	74.6 (T)	-	34	3.17<br>-	90 after 28 days	86.8 and 5-200	N/A	No		A 4 layered cell-sheet was used a control group. Compared to the control, the test scaffold at various concentrations, cell viability was not significantly altered.	Only the PLLA membranes were tested, and all four were independently assessed. There were slight variations in response for each membrane, but generally the same stress/strain curve was presented for each. The membrane moduli fall far below that of native bone tissue.
Kilian, et al. (2022) [147]	CPC+PCL	MEW and 3D printing	Bone	Compression<br>-<br>10 x 10 x (2-3)	~30-85 (C)	-	-	~2-11<br>-	-	-<br>457-1356	N/A	No		Generally, MSCs proliferated better across all pore size variations (800, 1200, and 1600) than mOBs, as the actin was better able to 'bridge' the gap.	Inclusion of PCL layers to the scaffold typically decreased modulus and yield strength performance across pore size. It was theorised by the group that this was due to interference in the setting of the CPC layers.
Liu, et al. (2022) [148]	PCL/Gel +PCL/Gel/HA+PCL/Gel/ Hep	Electrospinning and 3D printing	Bone	Compression<br>0.5 mm/min<br>12 × 12 × 10	5.96-13.86 (C)	-	-	-<br>-	-	54-60 and 500-600	N/A	Yes	The composite scaffold showed good integrative and regenerative properties, and displayed no cytotoxicity, while also acting a barrier to prevent infiltration by fibrous connective tissue. The bilayer scaffold demonstrated much greater new bone tissue formation, however.	The upper membrane (PCL/Gel/Hep) showed improved cell proliferation compared to PCL/Gel alone. Out of the 'lower' layers, PCL/Gel/HA showed higher cell count than PCL and PCL/Gel layers.	The PCL/Gel construct offered an increased modulus compared to pure PCL, however, this in turn was outperformed by the PCL/Gel/HA layer.
Chi, et al. (2022) [160]	PLA/ PDA/HA	3D printing	Bone	Tensile and Compression<br>1 mm/min (tensile)<br><br>5 mm/min (compressive)<br>10 x 10 x 3	126.9-168.1 (T)<br>30.7-39.2 (C)	-	-	-<br>-	98-94 after 28 days	36 and 400	N/A	No		N/A	Surface modification of the scaffolds, while generally seen to observe the scaffold modulus between groups with increasing HA content, did not do so in a statistically significant way.

Peng, et al. (2022) [161]	SF/OCP/ PDA	Freeze-drying	Bone	Compression<br>2 mm/min<br>Ø 10<br>L 10	0.043-0.066 (C)	-	-	-<br>-	93-73 after 35 days	75-81.2 and 136-143	850-1120	No		While no significant differences were observed, a general trend of increasing cell viability with increased PDA content was observed.	The SF-OCP scaffold provided a higher compressive strength than the SF and PDA-coated constructs. This was theorised to be due to the PDA disrupting the uniformity of the structure.
Aghajanian, et al. (2022) [64]	Forsterite + copper ferrite and Forsterite + copper ferrite/ P3HB	Sol-gel combustion	Bone	Compression<br>0.5 mm/min<br>10 x 10 x 10	2.2-4 (C)	-	-	-<br>-	99-94 (neutral buffer)<br>90-55 (citric acid)	73-86 and<br>2-5	N/A	Yes	Both scaffolds induced a positive response in terms of new tissue formation and trabecular thickness when compared to the control study However, the P3HB was observed to slightly increase these properties further. Additionally, neither specimen seemed to induce an immune response.	A significant increase in cell viability compared to the control and uncoated scaffold was noted. New bone tissue formation was also increased on the coated scaffold compared to the others.	Increasing the concentration of the P3HB coating (from 1-5% w/v) resulted in an increase in the moduli of each scaffold. The moduli obtained fall within the lower band of native tissue values, and thus are suitable for *in vitro* and *in vivo* applications.
Meng, et al. (2022) [170]	PLLA	MEW	Bone	Tension<br>10 mm/min<br>Ø 8<br>L 2	~1000-5000 (T)	-	~1.2-4.2	-<br>~20-75	~75-35% after 4 hours in ethanol	-<br>200	-	No		The modified PLLA scaffold demonstrated a large increase in tissue formation in comparison to the control PLLA construct.	Excessive NaOH concentration (0.5 M) and immersion time (1-4 hours) severely affected the mechanical properties of the scaffold. However, strain properties were unaffected with a 0.25 M concentration across all timepoints.
Mahendiran, et al. (2022) [166]	Palm dECM + silicon	Decellurising	Bone	Compression<br>1 mm/min<br>Ø 14<br>L 10	0.0006-0.008 (C)	-	-	-<br>-	~98-83% after 21 days	75.84-79.33 and 0.007-0.019	~1250-2500	Yes	Neither scaffold presented signs of inflammation nor infection. Both scaffolds exhibited neovascularisation, the presence of endothelial cells, and collagen network fibres. Quantification of these differences was not presented.	Compared to pure Palm dECM, modification through OTS enhanced cell proliferation, which was enhanced further by APTES treatment.	Pure dECM offered poor mechanical response; OTS treatment enhanced the modulus of the scaffold by several factors, with APTES increasing this value even further.
Feng, et al. (2022) [190]	β-TCP	3D printing	Bone	Compression<br>0.5 mm/ min<br>Ø 9.8<br>L 11	600-1750 (C)	-	~0.25-0.29	-<br>~10-39	-	33.4–43.8 and 1-1.5mm macropores	N/A	Yes	The capillary action effect promoted osteoinduction in the helical scaffold, as new tissue formation was noted throughout the scaffold.	The helical scaffold offered a significantly increased cell response compared to the grid-like pattern, with an increased pitch increasing cell proliferation further. This rise in cell growth was likely due to increased 'step' size on the spiral.	Compared to the grid scaffold, the presence of a helical structure increased the compressive strength of the design. Increasing pore size decreased the scaffold's compressive modulus.
Min, et al. (2022) [120]	Alginate (Core)/ Chitosan (Shell and gel)/ SF(gel)	Core-shell microspheres	Cartilage	Compression<br>10%/min<br>Ø 10<br>L 7-8	3-70 (C)	-	~14-65	-<br>0.02-0.06	-	-<br>-	N/A	No		Cell number initially grew slowly due to poor adhesion, then recovery followed at day 7. No significant differences in cell count between hydrogel mixtures.	Excessive variable factors make correlation difficult, generally increasing levels of genipin and/or tyrosinase crosslinkers increased the mechanical strength of the hydrogel.
Wang, et al. (2022) [113]	GelMA+ HyAMA + chondrospheroids	Hydrogel solution	Cartilage	Compression<br>1 mm/min<br>-	0.025-0.04 (C)	-	-	-<br>-	-	69-85<br>-	-	Yes	The chondro-spheroids maintained their morphology during the study. Genes COL 2, SOX 9, and HIF-1a were upregulated in comparison to the positive control (natural cartilage), while COL 10 was downregulated by comparison.	Cells proliferated rapidly, especially after one month *in vivo.*	HAMA and GelMA/HAMA hydrogels offered an increased, albeit similar, compressive modulus in comparison to GelMA alone.
Singha, et al. (2022) [116]	PEC/SF/ SA/PrP	Phase separation	Cartilage	Compression<br>0.5 mm/s<br>Ø 10<br>L 10	0.13 (C)	-	-	-<br>0.03-0.1	Complete degradation after 32 weeks	77 and<br>55–261	36-54%	No		PEC/SF/SA increased cell number, but cells grew slowly. Inclusion of PrP into scaffold greatly increased cell number and proliferation.	PEC/SF/SA roughly doubled the mechanical strength of the scaffold compared to PEC/SF alone. Inclusion of PrP increased this value further.
Semitela, et al.	PCL+Gelatin and	Electrospinning	Cartilage	Compression	0.44-7.85 (T)	-	-	-<br>-	85-70 after 6 days	4-6<br>-	N/A	No		Increasing concentrations of Gelatin resulted in a minor relative increase	An increase in Young's moduli was observed for both

(2021) [137]	PCL +Alginate			10 mm/min 15 x 15										in cell number. A similar effect was observed for Alginate, however a pronounced increase in cell number was observed.	scaffolds with increasing concentrations of Gelatin and Alginate. However, the effect of the Alginate layer generally offered an increased modulus in comparison to Gelatin.
Janarthanan, et al. (2022) [138]	Alg+HA (~70 layers)	Bioprinting	Cartilage	Compression - 10 x 10	0.013 (C)	-	-	- -	50-37 after 50 days	65-77 and 152-190	587-1170	Yes	No significant differences were found, in terms of integration, between 50:50 and 70:30 ratios of Alg + HA, however both scaffolds demonstrated high rates expression of macrophage F4/80 and angiogenesis protein CD31 compared to the control solution.	Alginate, HyA, and various combinations thereof were assessed. While no significant differences were observed, a minor trend of increasing cell viability with increased HyA content was noted.	Only one sample was tested, with a 50:50 ratio of Alginate to HyA. As cyclic loading was employed, a hysteresis loop was noted at each strain condition (25-50%).
Chen, et al. (2022) [122]	Gellan/ Alginate/ Thixotropic magnesium phosphate (TMP)	3D Printing	Cartilage /Bone	Compression 0.5 mm/min Ø 20 L 8	0.1-10 (C)	~1.6-1200 and ~1.2-100	-	- -	~20% to complete degradation after 8 weeks	48-80 and 100-210	80-100%	Yes	After 6 weeks of implantation, subchondral bone growth was slightly diminished in the alginate + gellan growth, and significantly higher in the TMP-BG group, compared to the control. By week 12, the alginate + gellan group showed improved subchondral bone growth compared to the control, and the TMP group showed further enhanced proliferation.	SA-GG scaffold significantly reduced cell number compared to control TCP. TMP content did not significantly affect this number.	Introduction of TMP to SA-GG scaffold significantly reduced stiffness and stress properties. Increasing levels of TMP reduced these further. Elasticity was affected in a similar way with increasing TMP levels.
Baskapan, et al. (2022) [123]	PCL/ Laminin/ Span80™ emulsion	Electrospinning	Kidney	Tensile 10%/min L 20 W 5	~0.02-0.063 (T)	-	~750-1000	- 0.011-0.021	-	- -	N/A	No		Poor cell growth with additions of laminin and emulsion until week 3 in comparison to pure PCL.	PCL/Laminin blend offered greatest mechanical strength, while the PCL and emulsion blends were largely similar. Performance of all scaffolds dropped at higher strain bands. Mechanical properties fell within range of some kidney tissues.
Xu, et al. (2022) [124]	PLA/Col	Electrospinning	Neural	Tensile - -	0.225-6.5 (T)	-	20-128	0.1-8.8 and 0.12-10	-	48-59 and 0.04-0.2	N/A	No		Introduction of Col to PCL scaffold marginally increased cell count, however at highest Col content cell number remained unaltered. Fibre alignment did not seem to influence cell proliferation behaviour.	Various fibre alignments and Col ratios were experimented with for the membrane. It was found that PCL with smooth, aligned fibres tested in a parallel direction offered the highest strength response, with increasing quantities of Col the stress but not affecting elongation at break. In the vertical direction, little significant difference was found between ratios and alignments.
Sang, et al (2022) [127].	Chitsosan/PEG/MWCNTs	Solution only	Neural	Compression 0.1 mm/min -	$0.79x10^{-3}$-$2.09x10^{-3}$ (C)	-	-	- -	82.3%-57.2% after 24 days	83-96 -	700-1700%	No		No significant differences were observed in cell proliferation rate between scaffold types. Increasing MWCNT content appeared to slightly increased cell adhesion.	Increasing MWCNT content (1-5%) induced a proportional increase in Young's moduli.
Habibizadeh, et al. (2022) [162]	PCL/ Chitosan / Alginate + MSCs	Electrospinning	Neural	Tension 10 mm/min 20 x 20	53.01 (T)	-	27	0.22 and 0.7	95% after 60 days *in vitro*	91.9 and 20	220	Yes	The PCL + Chitosan sheet showed a moderate inflammatory response and rapid degradation in comparison to the PCL + Chitosan + Alginate construct, which induced a mild inflammatory response and featured a slower degradation rate.	With TCPoly serving as the control, PCL/Chitosan offered a slightly reduced cell count by comparison. The addition of the Alginate microlayer increased this value past control levels, and the introduction of MSCs increased this value further.	Only the PCL/Chitosan combination was assessed. The mechanical properties of the engineered tissue exceed those of native tissue. However, as the focus of this study was cell behaviour with various surface modification treatments, this parameter is

															likely to be explored in further study.
DiCerbo, et al. (2022) [157]	PCL/ GelMA	PDMS mould UV curing	Osteochondral	Compression<br>10%/min<br>Ø 8<br>L 2	0.007-0.57 (C)	-	-	-<br>-	-	-<br>280-430	-	No		Variations in scaffold pore size did not lead to any significant differences in cell viability.	Increasing the pore size of both the pure PCL scaffold and the composite significantly decreased the modulus of both constructs. In addition, the strength of the composite scaffold was proportionally lower than that of the pure PCL for each respective pore size.
Yang, et al. (2021) [135]	Ti/Col + PLGA	3D printing/freeze-drying	Osteochondral	Compression<br>0.8 mm/min<br>Ø 4<br>L 4-2	1.46-73 (C)	-	-	-<br>-	Col+PLGA scaffold completely degraded in 12 weeks	59.08<br>and<br>30–100	N/A	Yes	The Col-PLGA group, while indicated superior cell proliferation at the defect site after 24 weeks, this was mostly just fibrous tissue. By comparison, the bilayered scaffold showed more new bone tissue and better integration with the host. The defect did not fully heal after 24 weeks for either construct.	Ti scaffold generally boasted higher cell count than Col + PLGA layer; bilayer graft as a whole showed significant levels of new bone tissue formation at 4-, 12-, and 24-week timepoints.	Ti scaffold vastly outperformed Col + PLGA layer. The cartilage values are closely representative of native tissue.
Tamaddon et al. (2022) [139]	Ti/PLA/ Col + PLGA and Collagen + HA	3D printing	Osteochondral	Compression<br>0.5 mm/min<br>Ø 7<br>L 7	1.28-0.37x10^3 (C)	-	-	0.21-63<br>-	-	500-750<br>-	N/A	Yes	The multi-layer scaffold provided a more homogenous response in terms of 'filling in' the defect. In addition, the Col + HA scaffold was rougher than the multi-layer, featuring cracks and fissures. In general, the multi-layer scaffold offered a more complete repair response.	Ti scaffold demonstrated largely increased cell count and proliferation speed compared to the PLA and Col+PLGA scaffolds. This was due to the increased surface area of the Ti scaffold.	Only the individual layers of the multi-layer scaffold were mechanical tested. Ti scaffold boasted highest strength, followed by the PLA scaffold, then the Col+PLGA. A similar result was obtained for the yield strength analysis.
Rashidi, et al. (2021) [140]	PCS/Col + HA	Freeze-drying	Osteochondral	Dynamic<br>0.1–1001 s^{-1}<br>-	0.0085-0.071 (C)	-	-	-<br>-	90% after 14 days	97.6-98.7<br>and<br>33-117	N/A	No		In general, larger quantities of CS in the scaffold yielded greater cell proliferation over a 21 day period, with the HA and bilayer scaffolds offering a diminished cell number by comparison.	The bilayer scaffold was not tested. However, as this was formed from the optimal chondrogenic and osteogenic layers, it can be assumed that a more positive strength response would have been obtained in comparison to the individual layers. The HA scaffold offered the greatest modulus out of each layer tested.
Dargoush et al. (2022) [134]	PCL + PEO + PES and PCL + PEO/rGO + HA-Sr + PES-BH$_{0.5\%}$	Electrospinning	Osteochondral	Tensile<br>5 mm/min<br>-	0.018-0.078 (T)	-	-	-<br>4	-	-<br>-	N/A	Yes	The nanocomposite scaffold showed a larger upregulation in COL II, COL X, SOX 9, ALP, and Osteocalcin genes and protein compared to the hybrid scaffold. The hybrid scaffold may have induced an immune response due to degradation.	The rGO + HA-Sr/PCLPEO scaffold presented a statistically significant increase in cell viability after 5 and 7 day timepoints compared to just PCL/PEO alone.	In inclusion of the HA-Sr layer dramatically increased the tensile modulus of the scaffold. The UTS was largely unaffected, however. This suggests a diminished elasticity in the nanocomposite-based scaffold, but is not possible to explore further as a histogram was used to illustrate these values.
Wu et al. (2021) [133]	Silk/SilMA+ Sucrose	Hydrogel solution	Osteochondral	Compression and shear<br>15 mm/min and 2 mm/min (shear)<br>Ø 10<br>L 6<br>and	1-10.1 (C)	-	39-52<br>25-39 (shear)	2-9<br>-	-	-<br>300–500	-	Yes	The Silk and BMP-2 + Silk integrated poorly, however the latter of these did promote large volumes of new bone tissue. Comparatively, the bilayer scaffold alone showed very little new tissue formation. The BMP-2 + bilayer integrated and promoted new tissue growth well, superseded only by the Silk + SilMA composite scaffold.	TGF-β3 was introduced to the scaffold, which results in a large increase in cell number across two unspecified timepoints.	Increasing concentrations of GelMA increased the modulus of the scaffolds under compression and shear conditions. The dense scaffold far outperformed the porous in this regard.

				Ø 1<br>L 1 (shear)											
Li, et al. (2022) [141]	GelMA+ PEO/GelMA + PEO + HA	Hydrogel solution	Osteochondral	Compressive and dynamic 40mm amplitude 0.1 to 100 s^{-1} (shear)<br>Ø 8.5<br>L 3	0.004-0.016 (C)	3-14 and loss modulus not studied	-	-<br>-	-	-<br>1-325	380-642	Yes	It was stated and illustrated that the tri-layer scaffold demonstrated good capacity for regenerating cartilage, subchondral bone and trabecular bone. These properties were not quantified or examined further, however.	Introduction of an HA layer demonstrated increased cell viability at increasing ratios of GelMA to PEO. Results were largely similar without HA, however.	The effect of an HA layer was untested in compression. However, dynamic analysis suggested an approximate doubling of mechanical strength compared to the non-HA hydrogel.
Browe et al. (2022) [156]	AC-ECM/ Bone-ECM	Freeze-drying	Osteochondral	Compression<br>-<br>Ø 4<br>L 4	1.2-1.4 (C)	-		-<br>-		-<br>45-140	N/A	Yes	Broad variation in defect repair quality was found. Generally, however, the bilayered scaffold promoted zonally defined tissue, and was able to return the mechanical properties of the region close to that of the surrounding osseous region. Bone repair was more consistent than that of the natural healing process	No significant difference was found in DNA or collagen levels, but a large increase in sGAG expression was obtained in the AC-ECM scaffold.	No significant difference between moduli for standard or annealed scaffolds was obtained. However, it is important to note that the values obtained fall below those of native tissue.
Nejad et al. (2021) [142]	PCL/BG and PCL/HyA	3D printing	Periodontal	Compression<br>1 mm/min<br>Ø 5<br>L 2	51.6-67.4 (C) and 0.8-1 (C)	-	0.5, 0.59	~5, ~8 and 22.4, 22.5	-	-<br>200-300	-	No		No significant changes in cell viability were observed between scaffold types.	The inclusion of BG significantly increased the modulus of the scaffold compared to the Hya-based construct. Interestingly, yield and UTS values were similar for each scaffold, while strain performance was largely unaffected.
Salehi, et al.(2022) [125]	PCL/SF/ SESM/Gel. and PCL/SF/ SESM/MC	Electrospinning	Skin	Tensile<br>5 mm/min<br>10 × 50	3.46-18.43 (T)	-	24-38	-<br>-	82% after 28 days	-<br>-	-	No		Both PCL/SF/ SESM/Gel. and PCL/SF/ SESM/MC scaffolds showed increased, yet similar, increases in cell viability after 14 days.	The PCL/SF/ SESM/MC scaffold provided a significantly increased stress/strain response over the PCL/SF/ SESM/Gel.
Li et al. (2021) [126]	PCL/ Col/ZnO	Electrospinning	Skin	Tensile<br>-<br>-	0.43-2.05 (T)	-	1.48-94.31	0.4-2.2<br>-	48.98% after 15 days	55-82<br>-	80-430%	Yes	Gross imaging and linked diagram indicate that wound healing rate was enhanced by use of PCL + Col, and further enhanced by inclusion of ZnO, before reaching its highest rate with inclusion of Zno + VEGF. A similar trend was noted during Col and TGF-β1 expression analysis.	Inclusion of both Col alone, as well as ZnO, significantly improved cell viability compared to pure PCL scaffold. No significant differences between the modified scaffolds were present.	Different ratios of Col to PCL were tested. It was found that a 2:1 ratio of Col to PCL improved the overall strength of scaffold compared to pure PCL, while the tensile strength between the two was largely similar. Increasing ratios of Col to PCL diminished these characteristics significantly.
Zhong, et al. (2022) [163]	PCL/PEG/ PCEC/Hydrogel	Electrospinning	Skin	Compression<br>10 mm/min<br>Ø 10<br>L 5	0.001-0.01 (C)	1-10 and 0.3-1.9	-	-<br>-	-	-<br>-	-	No		N/A	The modified PCL fibres, once introduced to the hydrogel, increased the elastic modulus of the construct by a factor of 10.
Sheikhi, et al. (2022) [159]	PCL/ Chitosan / Gelatin	Electrospinning	Skin	Tensile<br>-<br>50 x 10	20.2-62.5 (T)	-	5.25-25.7	-<br>0.89-4.99	-	-<br>-	75-160	No		No significant differences were observed between the control and grafted scaffolds.	A significant decrease in UTS, modulus, and strain values was recorded with increasing quantities of grafted collagen.
Dorishettya, et al. (2022)	RSF and RSF/rGO	Electrospinning	Unstated	Nanoindentation	0.27-1.77 (C)	~3-17 and ~0.3-3.4	-	-<br>-	-	-<br>and	N/A	No		No significant change found, however a small correlation between increasing rGO content and	Increasing quantities of rGO induced large changes in Young's modulus compared

[121]			- uses for cartilage	0.183 N/m @ 20nm tip diameter<br>Ø 20<br>L 2						11.36-15.36				decreasing cell number was observed.	to RSF alone. Strain performance was also significantly decreased for increasing rGO content.
Wang, et al. (2022) [164]	PCL/ ePTFE/ RGD	Material/ solvent solution	Vascular	Tension<br>3 mm/min<br>40 x 10	49-687 (T)	-	275-300	~6-8 and 9.5-16	-	-<br>-	N/A	No		Inclusion of PCL surface modification and RGD coating to ePTFE surface induced a significant increase in cell viability compared to pure ePTFE.	Increasing the PCL wt% induced a corresponding increase in modulus and UTS values. The yield stress values were not necessarily proportionate however, with the 1.3wt% PCL scaffold offering a lower yield strength that the other two composite constructs.
Xiao, et al. (2022) [165]	PCL + PGS + DTβ4	Electrospinning and 3D printing	Vascular	Tension<br>5 mm/min<br>Ø 2.4<br>L 5	170-710 (C)	-	34.9-58.4	-<br>102-247	70-15 after 5 hours	29.1-70.1<br>-	N/A	Yes	Patency rate for scaffold was maintained at 80% across test animals, showing no signs of dilation or thrombosis. However, the grafts degraded before native tissue could remodel around the grafts. Slight generation of cross-linked elastin was noted, as well as rapid endothelialisation.	Introduction of DTβ4 peptide significantly increased cell proliferation.	Increasing the PGS component resulted in an increased modulus and significantly increased UTS; however, the strain values were in turn decreased, due to increasing stiffness of the scaffold.
Bazgir, et al. (2021) [143]	PCL+PLGA (coaxial) and PLGA/ PCL	Electrospinning	Vascular	Tensile<br>0.5 mm/s<br>35 × 6	33.84 (T) and 7.4 (thickest tested scaffolds) (T)	-	42 and 18	5.39, 2.94<br>-	95 for both	35-40 and 56.03, 20.16	-	No		N/A	A series of scaffold thicknesses were tested. It was found that, generally, the coaxial scaffolds outperformed the bilayer constructs in almost every mechanical aspect. The thicker scaffolds also typically produced a higher modulus.
Li, et al. (2021) [144]	PCL/PCL	Electrospinning	Vascular	Tensile<br>1mm/min<br>Ø 4<br>L unspecified	30.69 (T)	-	190	10.34 and 191.2	-	-<br>-	-	Yes		N/A	The mechanical properties of the scaffold closely resemble that native human coronary arteries.
Do et al. (2021) [145]	RHC+ PCL/PEO+PCL	Electrospinning	Vascular	Tensile<br>2 mm/min<br>4 x 5	2.16-4.92 (T)	-	74.83-116.07	-<br>4.28-8.47	96-83 after 30 days	44.29-76.85 and 7.76-27.9	865-989	No		No statistical difference was observed between cell and/or scaffold types after 7 days, however cells seemed to proliferate well across all scaffold combinations.	No linear trends were observed in terms of PCL to RHC ratio and mechanical performance; however, generally a larger quantity of PCL than RHC would lead to a stronger scaffold overall. The outer layer solution of PCL + PEO quantities were kept constant for each scaffold.
Smith, et al. (2022) [146]	dECM/HAfoam/dECM/dECM	Decellularising and gas foaming	Vascular	Tensile<br>25.4 mm/min<br>Ø 6<br>L 25	6.06-8.05 (T)	-	32.73-41.49	1.21-1.87<br>-	-	-<br>-	-	No		A DMEM + 10% FBS solution was included as a positive control for all studies, and outperformed all other material combination. This was followed by the composite scaffold, the GAGF layer, ddECM layer, and finally the media only solution.	3 samples were assessed; triple layered ddECM (OFM-3), 5 layered ddECM (OFM-5), and a composite of GAGF and ddECM. Of these, OFM-3 offered the highest yield and modulus performance, followed by the OFM-5 and CMP constructs. This appears to be due to the inclusion of the HA foam in the composite structure.
Thompson, et al.	EF/EF/EF/DS	Embedded 3D printing	Vocal cords	Tensile<br>-<br>-	0.009-0.04 (T)	-	-	-<br>-	-	-<br>-	-	No		N/A	The Dragonskin™ silicone layers vastly outperformed the EcoFlex™; however, the

(2021) [152]															focus of this study was to develop and oscillating vocal cord system with similar material properties to that of native tissue; the values obtained lie within native tissue range.
